# Parasitic Appendicitis: A Novel Laparoscopic Approach for the Prevention of Peritoneal Contamination

**DOI:** 10.1155/2018/3238061

**Published:** 2018-05-24

**Authors:** Elbrus Zarbaliyev, Sebahattin Celik

**Affiliations:** ^1^Department of General Surgery, Gaziosmanpaşa Hospital, Yeni Yüzyil University, İstanbul, Turkey; ^2^Department of General Surgery, Faculty of Medicine, Yuzuncu University, Van, Turkey

## Abstract

**Background/Aim:**

Although rare, parasitic infection can cause acute appendicitis and result in contamination of the peritonea during appendectomy. The goal of this study was to summarize our experiences with parasitic appendicitis and describe a novel laparoscopic technique to prevent contamination.

**Method:**

All patients diagnosed with acute appendicitis who underwent appendectomy between January 2016 and January 2017 were included in the study. All appendectomies were performed using the standard three-port laparoscopic method, and a video recording was made of each procedure. Following separation of the mesoappendix, a single endoloop was placed in the base of the appendix, and the appendix was then transected 3-4 mm above the clamp with the aid of a thermal cauterizing/sealing device. The appendix was extracted from the 10 mm trocar hole below the umbilicus and placed inside a bag prepared from a glove. After pathological confirmation of parasitic appendicitis, medical records were retrospectively analyzed in each case for whether peritoneal contamination had occurred or not.

**Results:**

Out of 97 appendectomies, parasitic infection was observed in 4 cases, as confirmed by pathological examination. In two of these patients, *E. vermicularis* was detected, while the other two were infected with *Balantidium coli*. Intraoperative contamination did not occur in any of the cases, and retrospective review of the video recordings indicated no peritoneal contamination.

**Conclusion:**

As a result of the coagulation and sealing effects of thermal devices, airtight seals were created on the residual appendiceal stumps, and consequently, no contamination was observed in any of the cases.

## 1. Introduction

Acute appendicitis is the most common reason for emergency surgery and the most common reason for surgery of the gastrointestinal system (GIS) [1]. The condition is usually caused by increased pressure within the lumen following its obstruction due to fecaloid matter, after which infection develops as a result of bacterial translocation. Fecaloids and viral infection are the most common causes of appendicitis, while tumors, inflammatory bowel diseases, and parasites rarely lead to this pathology [2]. The parasite that is commonly encountered following appendectomy is *E. vermicularis*. In addition, parasites such as *Entamoeba histolytica*, *Schistosoma* sp., *Taenia* sp., *Ascaris lumbricoides (Ascaris),* and very rarely, *Balantidium coli* have also been reported to cause appendicitis [3, 4]. Although the role of the parasites in the development of acute appendicitis has not yet been settled, parasites such as *E. vermicularis* and *Ascaris* have been reported to obstruct the appendix lumen, thus resulting in acute appendicitis [5].

While a definite connection between *E. vermicularis* and acute appendicitis has not yet been established, infestation with the former may present symptoms imitating acute appendicitis. *E. vermicularis* remains the parasite most responsible for appendicitis.

*Balantidium coli* is a unicellular parasite. Although it generally has an asymptomatic course, it has been shown to cause abdominal pain, dysenteric symptoms, cystitis, and pneumonia [6]. *B. coli* sp. has been reported to be a very rare cause of acute appendicitis [5].

Currently, laparoscopic appendectomy is the standard method of appendectomy performed. Following laparoscopic appendectomy, parasitic infestation may be detected. In some studies, parasites have been detected intraoperatively, and peritoneal contaminations have been reported [7, 8]. In our study, we retrospectively reviewed all the records of our cases of appendectomy with parasitic infection and, supplementing our data with findings reported in the literature, evaluated procedures to prevent parasitic peritoneal contamination.

## 2. Patients and Methods

All patients with a diagnosis of acute appendicitis who underwent appendectomy between January 2016 and January 2017 were retrospectively included in the study. The primary reason for selecting this time period was the fact that all the appendectomy surgeries beginning with the start date were performed by laparoscopy, with video recordings made of the procedures. By retrospectively analyzing the surgical records for the cases in which parasitic infection was observed based on the pathology results, the methods applied could be reviewed. The files, pathology results, and video recordings of the surgeries of patients diagnosed with parasitic infestations were retrospectively analyzed and reviewed.

## 3. Surgical Technique

**Figure 1:**
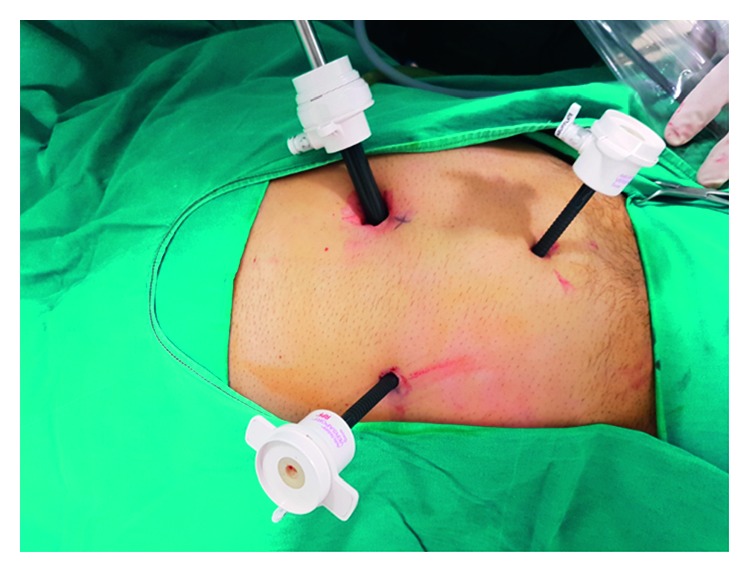
Placement of the trocar for laparoscopic appendectomy.

**Figure 2:**
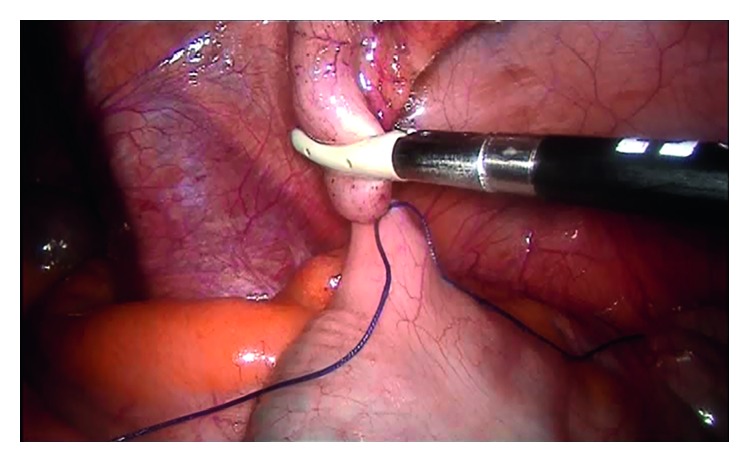
Separation of the appendix.

**Figure 3:**
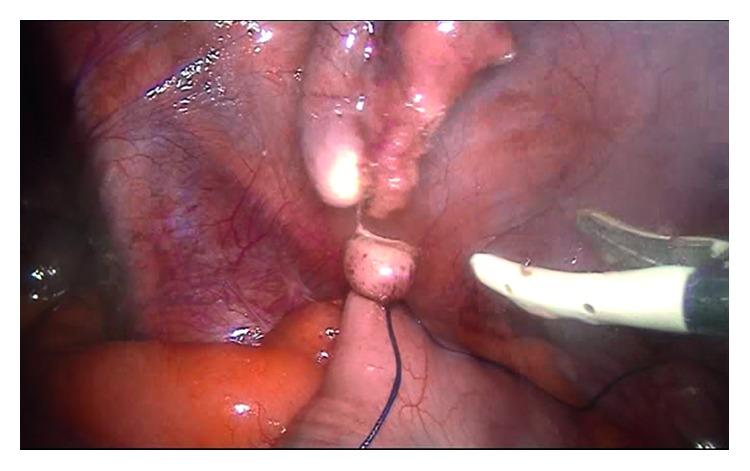
View of the residual appendix.

**Figure 4:**
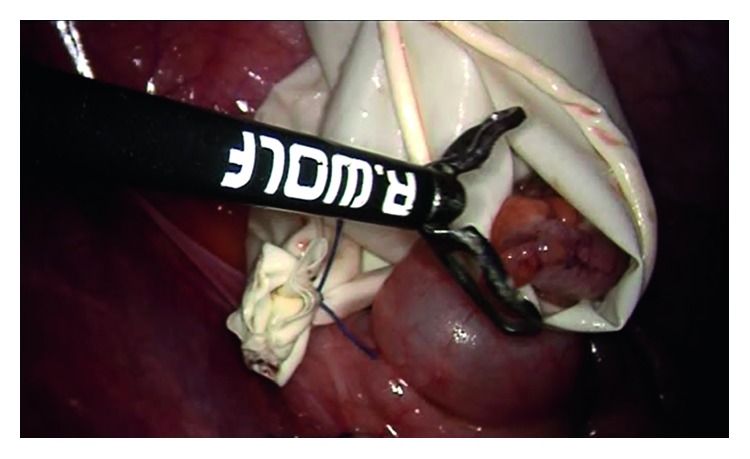
Removal of the appendix.

For all patients, surgery was performed laparoscopically using the standard three-port method (Figure 1), in which one 10 mm and two 5 mm trocars were employed. Following separation of the mesoappendix, a single endoloop was placed in the base of the appendix, and the appendix was transected 3-4 mm above the suture by means of a thermal cauterizing device. Specimens were then placed into a bag prepared from gloves and removed through the 10 mm trocar hole below the umbilicus (Figures 1–4).

## 4. Results

**Figure 5:**
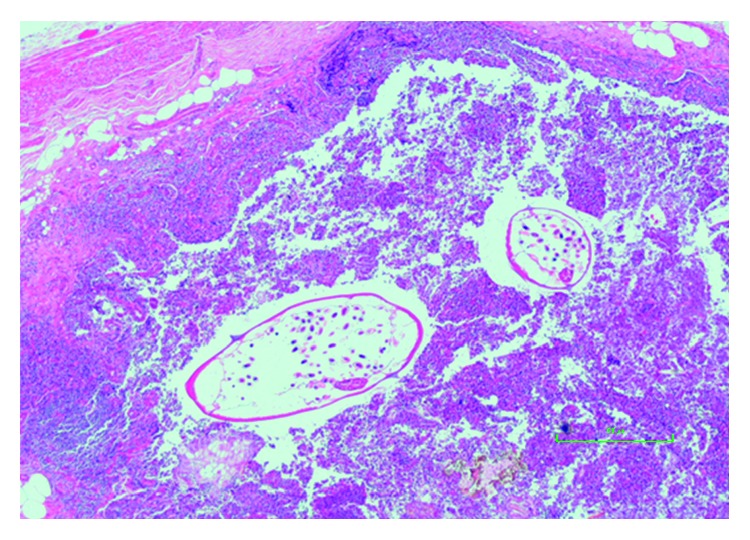
Appendix infested with *E. vermicularis*.

**Figure 6:**
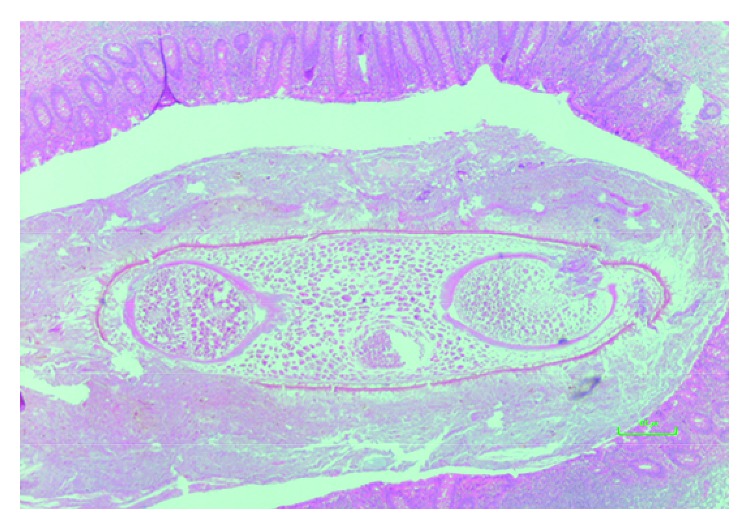
Appendix infested with *B. coli*.

Over the course of the one-year study period, a total of 97 patients underwent laparoscopic appendectomy surgery. In four (4%) patients, the pathology results indicated parasitic infection. *E. vermicularis* was detected in two of these patients, and *B. coli* infestation was detected in the other two (Figures 5 and 6, resp.). One patient who was diagnosed with *B. coli* was a pig farmer, while the others were not involved with animal husbandry. All patients were from the Marmara region in northwestern Turkey.

The Alvarado scoring system, commonly employed in the diagnosis of acute appendicitis, was used for all patients. The mean score for the 93 patients who did not have parasitic infestation was 6.75, while that of patients with parasitic infestation was 6.5. The average age of patients with infestation was 26.25. The average age of patients with *E.vermicularis* was 11.5 (11 and 12 years old), while those with *B. coli* had an average age of 41 (23 and 59 years old). Pathologically confirmed acute infection of the appendix and clinical fever (>38°C) were only detected in one of the patients infected with *B. coli*. Intraoperative contamination was not observed in any of the cases, and retrospective review of the video records of all cases found no peritoneal contamination. All patients were discharged within 18–20 hours after surgery. In postoperative follow-up of all patients, microbiological correlation was performed and medical treatments were begun.

## 5. Discussion

*E.vermicularis* is an intestinal parasite usually encountered in childhood and more often in female than in male children. It is primarily found in underdeveloped countries and in regions with lower socioeconomic levels [5]. As with many other gastrointestinal nematodes, pinworms do not require a vector for transmission. Pinworm infection usually occurs through ingestion of infectious eggs due to direct anus-to-mouth transfer via the fingers. This is facilitated by the perianal itch (pruritus ani), induced by the presence of pinworm eggs in the perianal folds, and commonly occurs as a result of nail biting, poor hygiene, or inadequate handwashing. One study reported that *E.vermicularis* infestation has different clinical presentations and that the parasite causes acute appendicitis [9]. Although *E.vermicularis* as a cause of acute appendicitis is still under debate, other studies have also concluded that it can cause acute appendicitis [10, 11]. Different rates of *E.vermicularis* infestation following appendectomy have been reported for Turkey and other countries [12–14]. In our study, the rate of *E.vermicularis* infestation was 2%. Although the diameters of the appendices in the two cases with *E.vermicularis* infestation were 6.5 mm and 6.8 mm based on abdominal ultrasonography (USG) results, there were no findings of acute infection, neither macroscopically nor microscopically. However, some studies have shown that acute infection accompanies *E.vermicularis* infestation. While mesenteric lymphadenitis was not detected in either patient, *E.vermicularis* is thought to cause mesenteric lymphadenitis.

Balantidiasis (also known as balantidiosis) is defined as infection of the large intestine with *B. coli*, a ciliated protozoan. *B. coli* are known to parasitize the colon, and pigs may be their primary reservoir. *B. coli* is a parasite that lives in domesticated (mostly pigs) and wild mammals and is the only parasite with cilia that can cause parasitic infection in humans. Its prevalence varies between 0.01 and 1% in different populations [15]. Contamination by this parasite, which lives in the distal ileum and cecum areas in trophozoite form, occurs in cystic form through the fecal-oral route [16]. The parasite that occurs in trophozoite form in the intestinal lumen penetrates the intestinal mucosa by excreting hyaluronidase enzyme and can cause ulcers. Cases resulting in mortality due to intestinal perforation and peritonitis have also been reported [17]. Some studies have reported *B. coli* as a cause of acute appendicitis [18]. *B. coli* infestation was detected in two of our cases. In one of those cases, macroscopic and microscopic findings of acute infection were observed in the appendix.

The rate of parasitic infection in our study was 4%. However, other studies have reported rates between 0.2 and 42% [19]. In cases of appendectomy accompanied by parasitic infection, microscopic and macroscopic findings of acute infection are generally not seen. In our study, 75% of parasitic infections did not indicate acute appendicitis. Although acute infection was not observed in these patients, there were other reasons to recommend appendectomy. As laparoscopic exploration is now possible, appendectomy is often preferred even in cases where the appendix is of normal appearance in order to treat recurrent appendix pains, and especially to reduce the potential for adnexal pathologies in female patients [20, 21]. Nonetheless, the merits of such a decision are debatable, as parasitic infestation is often seen in these cases. As a result, parasites such as *E.vermicularis* and *Ascaris* have been shown to present findings similar to appendicitis without acute infection [22, 23]. Therefore, another threat that the surgeon encounters in such cases is contamination of the peritoneal cavity by parasites existing in the appendix, which may or may not be seen macroscopically during the appendectomy. In such cases, the surgeon must determine the risk of parasitic infestation before surgery, based on the patient's medical history and standard clinical and/or laboratory tests performed prior to diagnosis of acute appendicitis. Peritoneal contamination has been reported in cases of parasitic infestation, particularly with *E.vermicularis*. Some studies advise cleaning the peritoneal cavity following the parasitic contamination of the peritoneal cavity and provide recommendations for preventing contamination [7]. Methods such as aspiration of parasites and cauterization of the parasites localized in the appendix stump are advised. The stapler application, which is considered effective, is nonetheless costly [23]. The challenges presented by parasitic infestation, such as the ability of *E.vermicularis* to attach to the mucosa and the fact that microscopic scale parasites such as *B. coli* cannot be seen macroscopically, must be kept in mind. We used a single endoloop in all our cases and transected the appendix 3-4 mm above the clamp with the aid of a thermal cauterizing device (Figure 6). In the retrospective review of surgery video recordings, the appendix stump was observed to have an airtight seal as a result of coagulation, and no contamination was detected in any of our cases. It should be recalled that risk of contamination occurs during extraction of the specimen. Although this risk is eliminated when the appendectomy is performed using the stapler method, it is a costly method requiring the use of a 12 mm trocar. The cut side and stump port of the appendix transected using the thermal method had airtight coagulation and the lumen was closed, preventing contamination (Figure 6). The appendix was extracted from the 10 mm trocar hole under the umbilicus and placed inside a bag prepared from a glove. Our patients were given antiparasitic (metronidazole) treatment.

In conclusion, clinicians should be aware that parasitic infestation may cause or simply mimic appendicitis. In both situations, peritoneal contamination may occur during appendectomy. Therefore, to prevent contamination, or at least minimize its risk, the use of thermal coagulation and sealing, which both kills the parasites and seals the appendix stump, is recommended.
